# Supplementary material for: Comparative Analysis of Conformational Dynamics and Systematic Characterization of Cryptic Pockets in the SARS-CoV-2 Omicron BA.2, BA.2.75 and XBB.1 Spike Complexes with the ACE2 Host Receptor: Confluence of Binding and Structural Plasticity in Mediating Networks of Conserved Allosteric Sites
Source: Viruses. 2023 Oct 10;15(10):2073. doi: 10.3390/v15102073 (PMC10612107; doi:10.3390/v15102073)
Supplement: Supplementary file 1 [file viruses-15-02073-s001.zip › SUPPLEMENTARY_MATERIALS_REVISION.pdf]

Supplementary Materials

Comparative Analysis of Conformational Dynamics and Systematic Characterization of Cryptic Pockets in the SARS-CoV-2 Omicron BA.2, BA.2.75 and XBB.1 Spike Complexes with the ACE2 Host Receptor: Confluence of Binding and Structural Plasticity in Mediating Networks of Conserved Allosteric Sites

Mohammed Alshahrani,[1] Grace Gupta,[1] Sian Xiao[2], Peng Tao[2], Gennady Verkhivker,[1,3]*

1 Keck Center for Science and Engineering, Graduate Program in Computational and Data Sciences, Schmid College of Science and Technology, Chapman University, Orange, CA 92866, USA; alshahrani@chapman.edu (M.A.); grgupta@chapman.edu (G.G.)
2 Department of Chemistry, Center for Research Computing, Center for Drug Discovery, Design, and Delivery (CD4), Southern Methodist University, Dallas, TX 75275, USA; sxiao@smu.edu (S.X.); ptao@smu.edu (P.T.)
3 Department of Biomedical and Pharmaceutical Sciences, Chapman University School of Pharmacy, Irvine, CA 92618, USA
* Correspondence: verkhivk@chapman.edu; Tel.: +1-714-516-4586

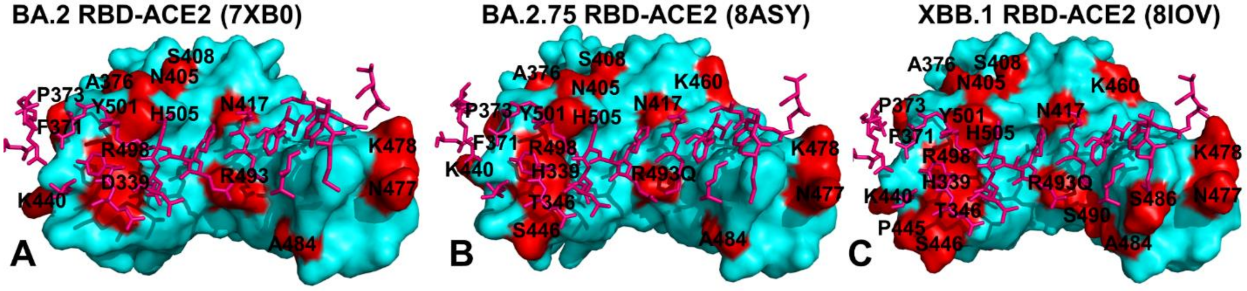

Figure S1. Structural organization of the SARS-CoV-2-RBD Omicron BA.2, BA.2.75, and XBB.1. binding interfaces in the complexes with human ACE2 enzyme. (A) The RBD-BA.2 is shown in cyan surface from the top view. The ACE2 binding residues are shown in pink sticks. The Omicron RBD BA.2 sites (G339D, S371F, S373P, S375F, T376A, D405N, R408S, K417N, N440K, S477N, T478K, E484A, Q493R, Q498R, N501Y, Y505H) are shown in red surface and annotated. (B) The RBD-BA.2.75 is shown from the top view. The ACE2 binding residues are shown in pink sticks. The Omicron RBD BA.2.75 sites (G339H, S371F, S373P, S375F, T376A, D405N, R408S, K417N, N440K, G446N, N460K, S477N, T478K, E484A, R493Q, Q498R, N501Y, Y505H) are shown in red surface and annotated. (C) The RBD-XBB.1 is shown from the top view and the ACE2 binding residues are shown in pink-colored sticks. The Omicron RBD XBB.1 sites (G339H, R346T, L368I, S371F, S373P, S375F, T376A, D405N, R408S, K417N, N440K, V445P, G446S,N460K, S477N, T478K, E484A, F486S, F490S, R493Q, Q498R, N501Y, Y505H) are shown in red-colored surface and annotated.

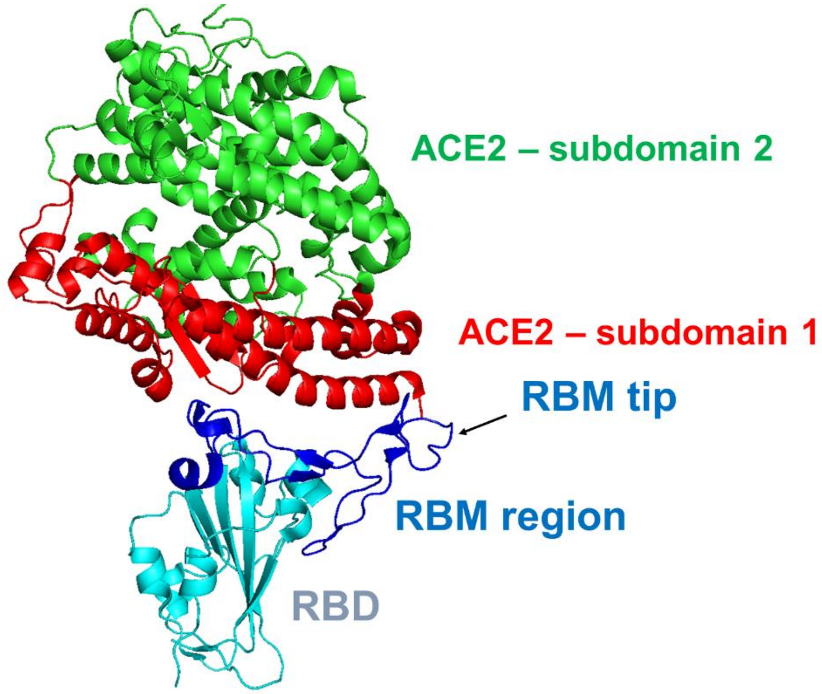

Figure S2. A general overview of the secondary structure elements and binding interface in the SARS-CoV-2 RBD complex with human ACE2. The RBD is shown in cyan and secondary structure elements are annotated. The RBM region is in blue ribbons and annotated. The subdomains I and II of human ACE2 are shown in red and green ribbons respectively.

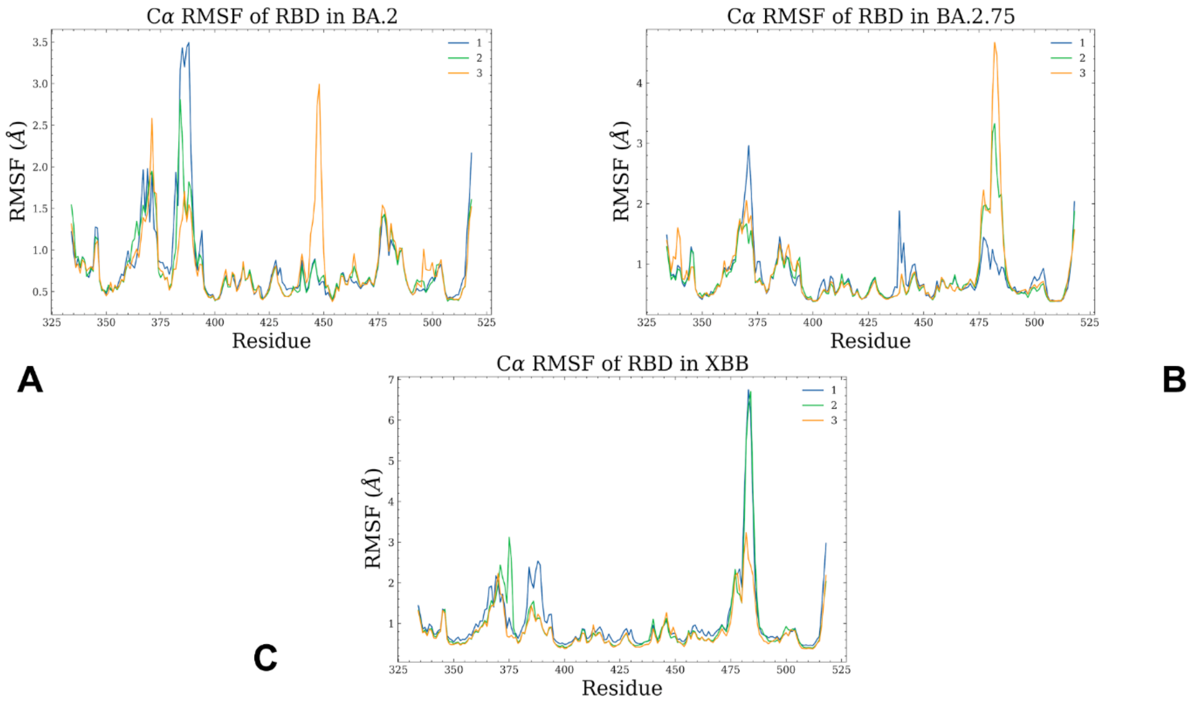

Figure S3. Conformational dynamics profiles of the RBD residues obtained from three independent MD simulations of the Omicron RBD BA.2, BA.2.75 and XBB.1 complexes with ACE2. The RMSF profiles for the RBD backbone residues obtained from three independent microsecond MD simulations of the Omicron BA.2 RBD-ACE2 (A), Omicron BA.2.75 RBD-ACE2 (B), Omicron XBB.1 RBD-ACE2 (C).

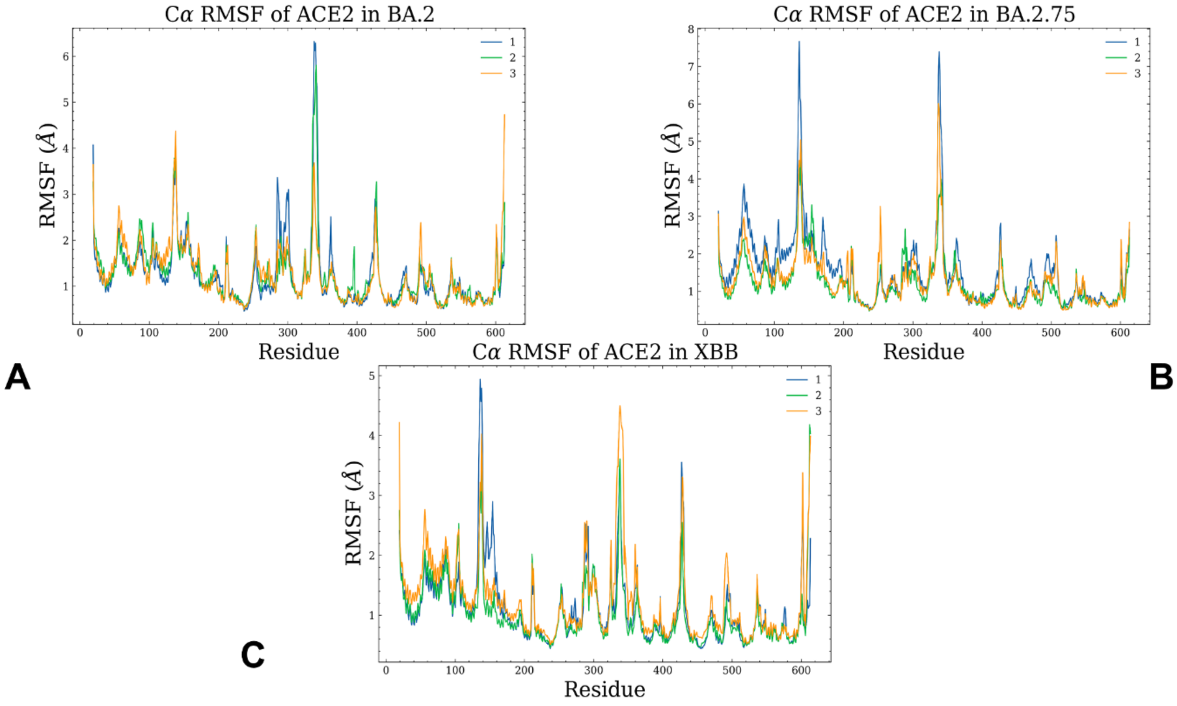

Figure S4. Conformational dynamics profiles of the ACE2 residues obtained from three independent MD simulations of the Omicron RBD BA.2, BA.2.75 and XBB.1 complexes with ACE2. The RMSF profiles for the ACE2 backbone residues obtained from three independent microsecond MD simulations of the Omicron BA.2 RBD-ACE2 (A), Omicron BA.2.75 RBD-ACE2 (B), Omicron XBB.1 RBD-ACE2 (C).

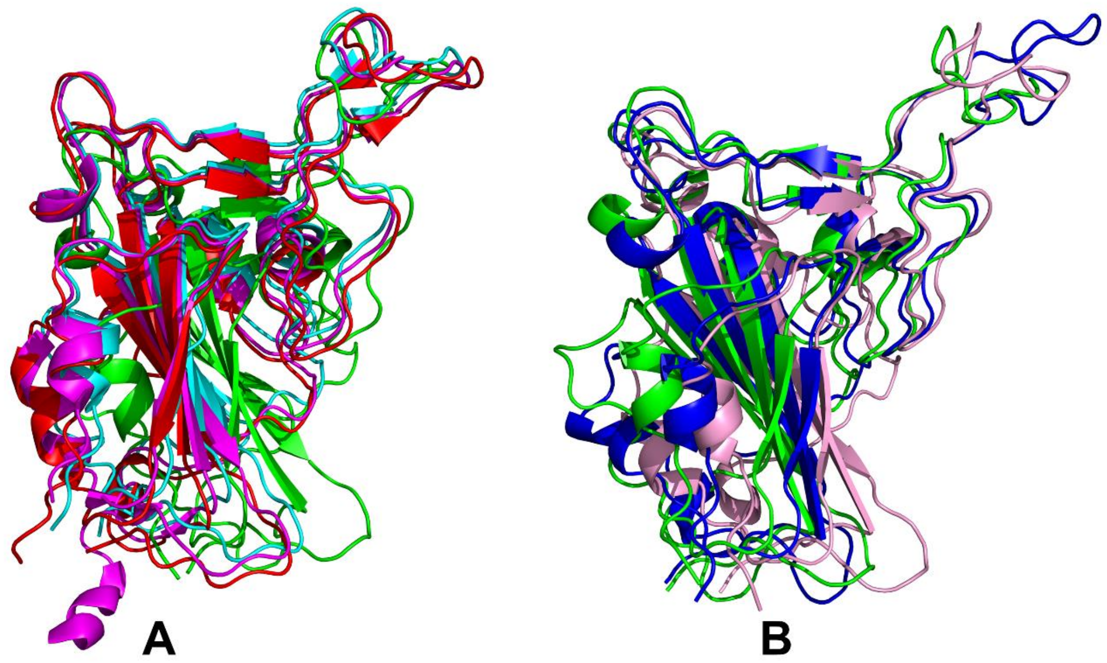

Figure S5. Structural analysis of the macrostates obtained from atomistic simulations of Omicron BA.2, BA.2.75 ad XBB.1 complexes with ACE2. (A) Structural alignment of the crystal structures of the Omicron BA.2 RBD (in cyan), BA.2.75 RBD (in magenta), XBB.1 RBD (in red) and RBD conformation of the macrostate 9 (in green). (B) Structural alignment of RBD conformations for macrostate 9 (in green), macrostate 8 (in pink) and macrostate 1 (in blue). The RBD structures are shown in ribbons. For clarity of the presentation the ACE2 molecules that are very similar in all structures and identified macrostates are omitted.

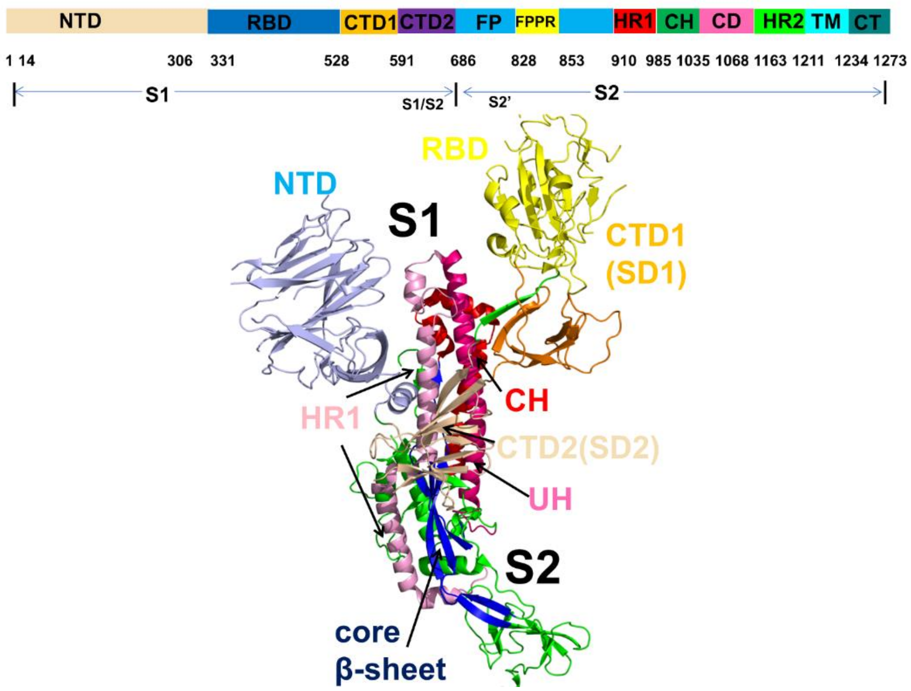

Figure S6. A schematic representation of domain organization and residue range for the full-length SARS-CoV-2 S protein. The subunits S1 and S2 include NTD RBD, C-terminal domain 1(CTD1), C-terminal domain 2 (CTD2), S1/S2 cleavage site (S1/S2), S2′ cleavage site (S2′), fusion peptide (FP), fusion peptide proximal region (FPPR), heptad repeat 1 (HR1), central helix region (CH), connector domain (CD), heptad repeat 2 (HR2), transmembrane domain (TM), and cytoplasmic tail (CT).The subunits S1 regions are annotated as follows : NTD (residues 14-306) in light blue; RBD (residues 331-528) in yellow; CTD1 (residues 528-591) in orange; CTD2 (residues 592-686) in wheat color ; upstream helix (UH) (residues 736-781) in red; HR1 (residues 910-985) in pink; CH (residues 986-1035) in hot pink; core β-sheet (residues 711-736, 1045-1076) (in blue).

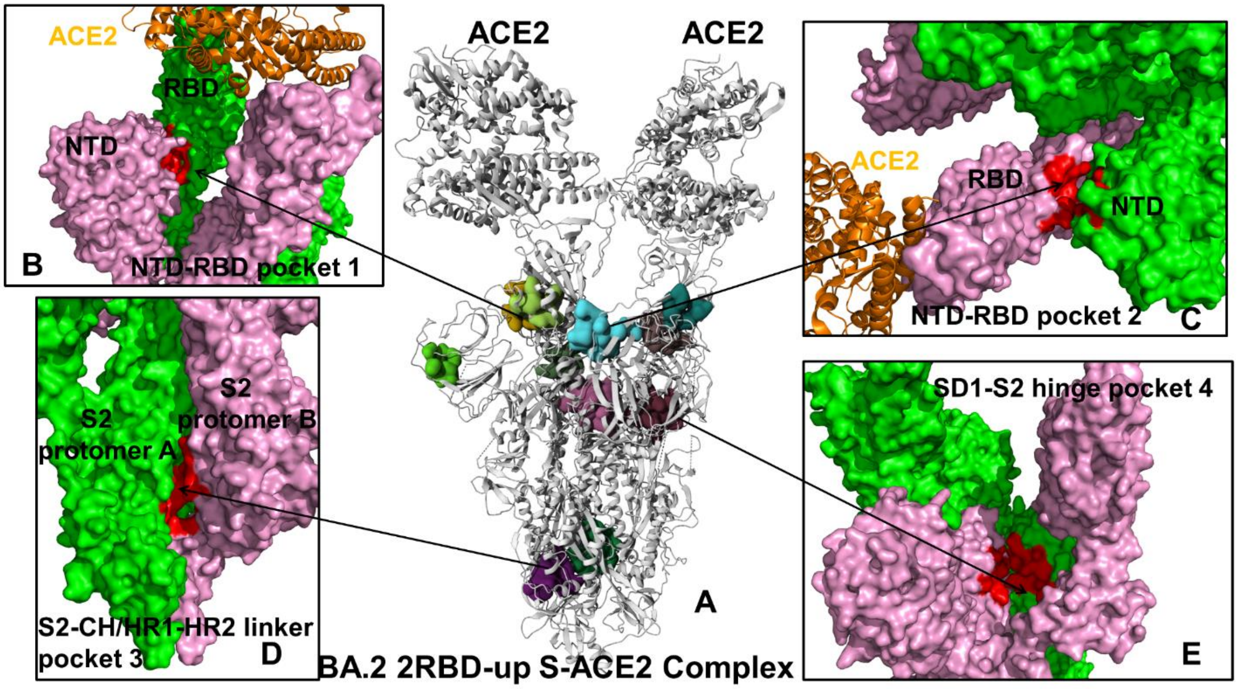

Figure S7. Structural map and residue-based close-ups of the top ranked cryptic sites for the ensemble of the S-BA.2 2RBD-up trimer complex with ACE2 . (A) The BA.2 2RBD-up trimer complex (pdb id 7XO7) is shown in ribbons and the top ranked allosteric sites are in surface. (B) Pocket 1 (NTD-RBD inter-protomer pocket, protomer A RBD residues A_357, A_359, A_360, A_393, A_394, A_520, A_521, A_523, and protomer C NTD residues C_115, C_130, C_168, C_230, C_231). (C) Pocket 2 (NTD-RBD interprotomer pocket, protomer B NTD residues B_115, B_132, B_167, B_168, B_170, B_230, B_231, B_232, and protomer C RBD residues C_357, C_393, C_394, C_518, C_520, C_521, C_523). (D) Pocket 3 (inter-protomer pocket in S2, protomer A residues A_1034, A_1035, A_1036, A_886, A_904, A_908, and protomer B residues B_1038, B_1039, B_1040, B_1046, B_1047, B_1107, B_1108, B_909). (E) Pocket 4 (inter-protomer pocket in S2, protomer A residues A_1002, A_970, A_995, A_998, A_999, and protomer B residues B_1001, B_1002, B_756, B_759, B_994, B_995, B_998, C_1002, C_756, C_970, C_994, C_995, C_998, C_999).
On panels (B-E) the S protein protomers are shown in green and pink surfaces. For clarity only two chains out of three protomers are highlighted. The predicted pockets are shown in red surface.

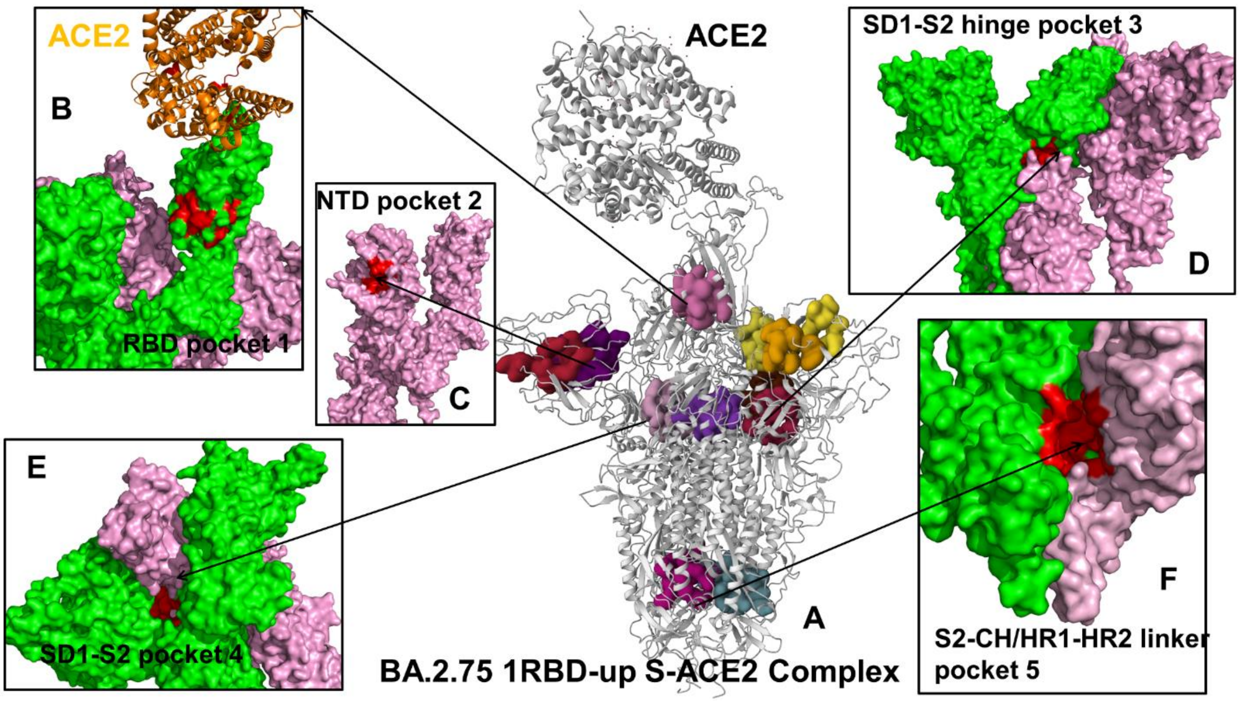

Figure S8. Structural map and residue-based close-ups of the top ranked cryptic sites for the ensemble of the S-BA.2.75 1RBD-up trimer complex with ACE2 . (A) The BA.2.75 1RBD-up trimer complex (pdb id 7YR2) is shown in ribbons and the top ranked allosteric sites are in surface. (B) Pocket 1 (RBD pocket residues on RBD-up protomer D: D_336, D_338, D_342, D_364, D_365, D_367, D_368, D_369, D_371, D_377, D_379, D_382, D_383, D_384, D_387, D_432, D_434, D_513, D_515). (C) Pocket 2 (NTD pocket residues on RBD-up protomer D: D_100, D_101, D_239, D_240, D_242, D_248, D_249, D_250, D_263, D_264, D_265, D_65, D_66, D_81, D_84, D_94, D_95, D_96). (D) Pocket 3 (inter-protomer SD1-S2 hinge pocket. Protomer C residues C_541, C_546, C_547, C_548, C_549, C_570, C_572, C_573, C_587, C_589, C_592, and protomer D residues D_1000, D_740, D_741, D_744, D_745, D_855, D_856, D_966, D_976, D_977, D_978). (E) Pocket 4 (inter-protomer SD1-S2 pocket. Protomer C residues C_974, C_976, C_979 and protomer E residues E_391, E_517, E_518, E_519, E_520, E_522, E_544, E_545, E_546, E_565, E_567, E_571, E_573).
(F) Pocket 5 (inter-protomer pocket in S2 CH/HR1-HR2. Protomer C residues C_1035, C_1036, C_1038, C_884, C_885, C_886, C_887, C_896, C_904, C_907, C_908, and protomer E residues E_1038, E_1040, E_1047, E_1107, E_1108, E_712, E_909, E_910). On panels (B-F) the S protein protomers are shown in green and pink surfaces. For clarity only two chains out of three protomers are highlighted. The predicted pockets are shown in red surface.

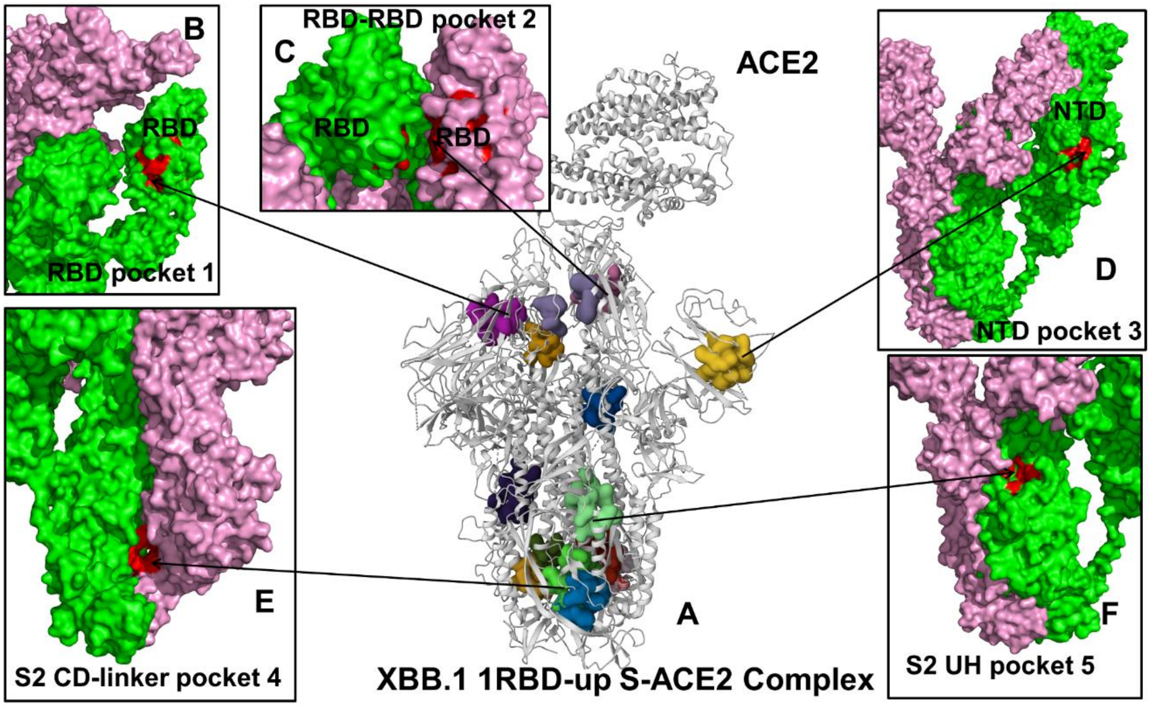

Figure S9. Structural map and residue-based close-ups of the top ranked cryptic sites for the ensemble of the S-XBB.1 1RBD-up trimer complex with ACE2 . (A) The XBB.1 1RBD-up trimer complex (pdb id 8IOU) is shown in ribbons and the top ranked allosteric sites are in surface. (B) Pocket 1 (RBD pocket residues on protomer C : C_338, C_365, C_368, C_369, C_377, C_387, C_432, C_434, C_513). (C) Pocket 2 (RBD-RBD pocket. RBD residues on protomer B B_369, B_372, B_373, B_374, B_377, and RBD residues on protomer C C_420, C_421, C_460). (D) Pocket 3 (NTD pocket on RBD-up protomer A : A_101, A_102, A_103, A_104, A_121, A_175, A_176, A_190, A_192, A_94, A_96, A_99). (E) Pocket 4 (inter-protomer S2 pocket in CD region of S2. Protomer A residues A_1034, A_1035, A_1036, A_886, and protomer B residues B_1038, B_1040, B_1047, B_1107, B_1108, B_909). (F) Pocket 5 (S2 pocket in UH region. Protomer A residues A_1056, A_1058, A_730, A_731, A_733, A_775, A_778, A_782, A_863, A_865, A_867, A_870).
On panels (B-F) the S protein protomers are shown in green and pink surfaces. For clarity only two chains out of three protomers are highlighted. The predicted pockets are shown in red surface.

Table S1. The list of the intermolecular contacts in the structure of SARS-CoV-2 BA.2 RBD-ACE2 complex (pdb id 7XB0).

ACE2 Residue	ACE2 Residue Number	ACE2 Chain ID	RBD Residue	RBD Residue Number	RBD Chain ID
ASP	355	A	GLY	502	B
TYR	83	A	ASN	487	B
GLN	24	A	ASN	477	B
ASP	355	A	THR	500	B
ASP	30	A	PHE	456	B
GLN	42	A	TYR	449	B
ASP	38	A	TYR	501	B
GLY	354	A	TYR	501	B
TYR	83	A	TYR	489	B
LEU	45	A	VAL	445	B
LEU	45	A	THR	500	B
LYS	353	A	TYR	495	B
GLN	24	A	PHE	486	B
HIS	34	A	LEU	455	B
LYS	353	A	GLY	502	B
LYS	353	A	THR	500	B
SER	19	A	ALA	475	B
LYS	31	A	LEU	455	B
LEU	79	A	PHE	486	B
GLN	24	A	TYR	489	B
TYR	41	A	ARG	498	B
ASN	330	A	THR	500	B
THR	20	A	ALA	475	B

LYS	353	A	VAL	503	B
ARG	357	A	THR	500	B
LYS	353	A	HIS	505	B
TYR	41	A	TYR	501	B
TYR	83	A	PHE	486	B
GLU	35	A	ARG	493	B
ASP	38	A	ARG	498	B
LEU	79	A	GLY	485	B
GLY	354	A	GLY	502	B
GLN	24	A	ALA	475	B
GLY	354	A	THR	500	B
ASP	38	A	GLY	496	B
PHE	28	A	TYR	489	B
ALA	25	A	ASN	487	B
THR	27	A	TYR	489	B
THR	27	A	PHE	456	B
SER	19	A	GLY	476	B
HIS	34	A	TYR	453	B
GLY	354	A	VAL	503	B
ASP	30	A	ASN	417	B
MET	82	A	PHE	486	B
GLU	37	A	HIS	505	B
GLY	354	A	HIS	505	B
LEU	45	A	ARG	498	B
ASP	38	A	TYR	449	B
THR	27	A	ALA	475	B

ASP	355	A	TYR	501	B
LYS	353	A	ARG	403	B
HIS	34	A	ARG	493	B
GLN	325	A	GLN	506	B
TYR	41	A	THR	500	B
HIS	34	A	ARG	403	B
GLN	24	A	GLY	476	B
GLY	352	A	GLY	502	B
GLN	24	A	ASN	487	B
GLN	42	A	ARG	498	B
LYS	31	A	ARG	493	B
ASP	38	A	TYR	495	B
SER	19	A	ASN	477	B
LYS	353	A	TYR	501	B
ASP	30	A	LEU	455	B
LYS	31	A	TYR	489	B
LYS	31	A	PHE	456	B
LYS	31	A	GLY	485	B
HIS	34	A	ASN	417	B
THR	27	A	TYR	473	B
THR	20	A	ASN	477	B
GLY	326	A	THR	500	B
GLN	42	A	GLY	446	B
GLY	352	A	TYR	501	B
ASN	330	A	PRO	499	B

Table S2. The list of the intermolecular contacts in the structure of SARS-CoV-2 BA.2.75 RBD-ACE2 complex (pdb id 8ASY).

ACE2 Residue	ACE2 Residue Number	ACE2 Chain ID	RBD Residue	RBD Residue Number	RBD Chain ID
ARG	357	A	THR	500	B
LYS	31	A	PHE	456	B
GLU	35	A	GLN	493	B
ASP	38	A	TYR	449	B
ASP	355	A	GLY	502	B
LYS	353	A	GLY	496	B
GLU	37	A	HIS	505	B
LEU	45	A	THR	500	B
THR	27	A	ALA	475	B
ASN	330	A	THR	500	B
TYR	83	A	TYR	489	B
ASP	30	A	PHE	456	B
THR	27	A	TYR	489	B
ASP	355	A	THR	500	B
LYS	353	A	TYR	495	B
HIS	34	A	GLN	493	B
PHE	28	A	TYR	489	B
PHE	28	A	ASN	487	B
ASP	38	A	GLY	496	B
SER	19	A	ASN	477	B
LYS	353	A	HIS	505	B
ASP	38	A	TYR	495	B

LYS	353	A	GLY	502	B
SER	19	A	ALA	475	B
THR	27	A	PHE	456	B
GLY	354	A	TYR	501	B
GLN	24	A	ASN	477	B
LEU	79	A	PHE	486	B
GLN	42	A	ARG	498	B
TYR	83	A	ASN	487	B
LYS	353	A	TYR	501	B
LYS	31	A	LEU	455	B
GLY	354	A	THR	500	B
LYS	353	A	THR	500	B
TYR	83	A	PHE	486	B
GLN	24	A	TYR	489	B
LEU	45	A	VAL	445	B
ASP	30	A	LEU	455	B
LYS	353	A	ARG	498	B
HIS	34	A	ASN	417	B
ASP	38	A	TYR	501	B
GLY	354	A	HIS	505	B
GLY	354	A	GLY	502	B
GLN	42	A	TYR	449	B
TYR	41	A	TYR	501	B
MET	82	A	PHE	486	B
HIS	34	A	TYR	453	B
GLY	352	A	TYR	501	B

ALA	25	A	ASN	487	B
TYR	41	A	THR	500	B
SER	19	A	GLY	476	B
LYS	31	A	TYR	489	B
LYS	31	A	GLN	493	B
GLY	354	A	VAL	503	B
ASP	355	A	TYR	501	B
ASP	38	A	ARG	498	B
TYR	41	A	ARG	498	B
LEU	45	A	ARG	498	B
GLN	24	A	ALA	475	B
GLN	24	A	GLY	476	B
GLN	42	A	SER	446	B
HIS	34	A	LEU	455	B
THR	27	A	TYR	473	B
GLN	24	A	ASN	487	B
LYS	31	A	PHE	490	B
GLN	325	A	VAL	503	B
GLN	24	A	PHE	486	B

Table S3. The list of the intermolecular contacts in the structure of SARS-CoV-2 XBB.1 RBD-ACE2 complex (pdb id 8IOV).

ACE2 Residue	ACE2 Residue Number	ACE2 Chain ID	RBD Residue	RBD Residue Number	RBD Chain ID
LEU	79	A	GLY	485	B
ALA	25	A	ASN	487	B
HIS	34	A	ARG	403	B
LEU	45	A	THR	500	B
GLN	24	A	ALA	475	B
TYR	83	A	ASN	487	B
ASN	330	A	THR	500	B
ASP	30	A	ASN	417	B
ASP	38	A	SER	494	B
TYR	41	A	ARG	498	B
GLN	42	A	ARG	498	B
TYR	83	A	TYR	489	B
GLY	354	A	GLY	502	B
LYS	31	A	SER	490	B
HIS	34	A	TYR	453	B
LYS	353	A	GLY	504	B
TYR	41	A	THR	500	B
THR	20	A	ASN	477	B
LEU	79	A	SER	486	B
THR	27	A	ALA	475	B
LYS	353	A	GLY	502	B
ALA	36	A	ARG	493	B
MET	82	A	ASN	487	B

HIS	34	A	ASN	417	B
GLY	326	A	THR	500	B
GLN	24	A	TYR	489	B
ASP	38	A	TYR	495	B
GLY	354	A	TYR	501	B
LYS	31	A	TYR	489	B
GLY	354	A	HIS	505	B
GLN	24	A	GLY	476	B
PHE	28	A	TYR	489	B
ASP	355	A	GLY	502	B
TYR	83	A	SER	486	B
ASP	38	A	ARG	498	B
ASN	330	A	PRO	499	B
GLN	42	A	TYR	449	B
LYS	353	A	TYR	501	B
HIS	34	A	SER	494	B
LEU	45	A	PRO	445	B
LYS	353	A	HIS	505	B
LYS	31	A	LEU	455	B
LYS	353	A	VAL	503	B
GLN	24	A	ASN	477	B
ASP	38	A	TYR	501	B
LEU	45	A	ARG	498	B
ASP	355	A	TYR	501	B
LYS	31	A	ALA	484	B
LEU	39	A	ARG	493	B

LYS	353	A	TYR	495	B
HIS	34	A	ARG	493	B
MET	82	A	SER	486	B
LYS	353	A	GLN	506	B
LYS	31	A	PHE	456	B
GLY	352	A	TYR	501	B
ASP	30	A	LEU	455	B
LYS	31	A	CYS	488	B
GLY	354	A	THR	500	B
SER	19	A	GLY	476	B
GLN	325	A	GLN	506	B
PHE	28	A	PHE	456	B
ASP	38	A	TYR	449	B
HIS	34	A	GLU	406	B
ASP	30	A	PHE	456	B
THR	27	A	TYR	473	B
TYR	41	A	TYR	501	B
GLN	42	A	SER	446	B
THR	27	A	PHE	456	B
SER	19	A	ASN	477	B
GLN	24	A	ASN	487	B
ASP	38	A	ARG	493	B
ARG	357	A	THR	500	B
HIS	34	A	LEU	455	B
GLU	35	A	ARG	493	B
GLU	37	A	HIS	505	B

THR	27	A	TYR	489	B
ASP	355	A	THR	500	B
SER	19	A	ALA	475	B
ASP	38	A	GLY	496	B
GLY	354	A	VAL	503	B
